# Comparative toxicity and biodistribution assessments in rats following subchronic oral exposure to copper nanoparticles and microparticles

**DOI:** 10.1186/s12989-016-0169-x

**Published:** 2016-10-28

**Authors:** In-Chul Lee, Je-Won Ko, Sung-Hyeuk Park, Na-Rae Shin, In-Sik Shin, Changjong Moon, Je-Hein Kim, Hyoung-Chin Kim, Jong-Choon Kim

**Affiliations:** 1 College of Veterinary Medicine BK21 Plus Project Team, Chonnam National University, Gwangju, 61186 Republic of Korea; 2 Natural Product Research Center, Korea Research Institute of Bioscience and Biotechnology, Jeongeup, 56212 Republic of Korea; 3 Gyeongnam Department of Environment & Toxicology, Korea Institute of Toxicology, Gyeongnam, 52834 Republic of Korea; 4 Laboratory Animal Resource Center, Korea Research Institute of Bioscience and Biotechnology, ChungBuk, 28116 Republic of Korea

**Keywords:** Copper nanoparticles, Copper microparticles, Comparative toxicity, Biodistribution

## Abstract

**Background:**

Copper nanoparticles (Cu NPs) have great potential in electronics and biomedical fields because of their efficient thermodynamic and anti-microbial properties. However, their potential toxic effects and kinetic data following repeated exposure are still unclear.

**Methods:**

We evaluated the physicochemical properties of Cu NPs (25 nm) and copper microparticles (Cu MPs, 14–25 μm). Comparative in vivo toxicity of Cu NPs and Cu MPs was evaluated by conducting a 28-day repeated oral dose study at equivalent dose levels of 0, 100, 200, and 400 mg/kg/day (vehicle, 1 % hydroxypropyl methylcellulose). We determined Cu levels in the blood, tissues, urine, and feces by using inductively coupled plasma mass spectrometry.

**Results:**

The solubility of Cu NPs and Cu MPs was 84.5 and 17.2 %, respectively, in an acidic milieu; however, they scarcely dissolved in vehicle or intestinal milieus. The specific surface area of Cu NPs and Cu MPs was determined to be 14.7 and 0.16 m^2^/g, respectively. Cu NPs exhibited a dose-dependent increase of Cu content in the blood and tested organs, with particularly high levels of Cu in the liver, kidney, and spleen. Only for liver and kidney increased Cu levels were found in Cu MPs-treated rats. Cu NPs caused a dose-related increase in Cu levels in urine, whereas Cu MPs did not affect the urine Cu levels. Extremely high levels of Cu were detected in the feces of Cu MPs-treated rats, whereas much lower levels were detected in the feces of Cu NPs-treated rats. A comparative in vivo toxicity study showed that Cu NPs caused damages to red blood cells, thymus, spleen, liver, and kidney at ≥200 mg/kg/days, but Cu MPs did not cause any adverse effects even at the highest dose.

**Conclusions:**

Overall, the in vivo repeated dose toxicity study of Cu NPs and Cu MPs demonstrated that large surface area and high solubility in physiological milieus could directly influence the toxicological responses and biodistribution of Cu particles when administered orally. Under these experimental conditions, the no-observed-adverse-effect levels of Cu NPs and Cu MPs were determined to be 100 and ≥400 mg/kg/day, respectively.

**Electronic supplementary material:**

The online version of this article (doi:10.1186/s12989-016-0169-x) contains supplementary material, which is available to authorized users.

## Background

Nanomaterials are defined as materials that have at least one dimension in the 1 to 100 nm range, which were generated by accidentally or engineering. The engineered nanoparticles (NPs) can be utilized in an application-specific manner by modifying their size, surface properties, and shape. Thus, in recent years, remarkable progress has been made in the area of nanotechnology as evident from its widespread use in textile, electronics, cosmetics, and foods [1]. The physicochemical properties of NPs may determinate toxicological behavior in vivo by making them interact with biological systems and be absorbed more than bulk chemicals via various routes [2, 3]. Hence, NPs tend to exhibit quite different toxicological profiles in vivo compared to the larger particles [4–7]. Physiological conditions also influence the interaction between biological systems and NPs and they can determine the fate and biosafety of NPs [8]. Recently, increased usage of NPs raises concerns on their health risks and environmental effects [9–11].

Copper (Cu) is an essential element required for normal physiological functioning, including drug/xenobiotic metabolism, carbohydrate metabolism, and the antioxidant defense system [12, 13]. The general population is exposed to Cu through inhalation, consumption of food and water, and dermal contact with air, water, and soil that contains Cu. The toxicity of Cu and its compounds has been studied for decades. A report of the available data has been given in the “Toxicological Profile for Copper” from the Agency for Toxic Substances and Disease Registry of the U.S. Public Health Service [14]. When intake of Cu exceeds the range of biological tolerance, it can cause adverse effects, including damage to liver, kidney, immune system, and gastrointestinal distress [14]. Although the toxic effects of Cu and its compounds have been studied, several studies reported gaps concerning the risk caused by Cu in the form of NPs [7, 15].

Among the various types of nanomaterials, metal-based NPs are used in the manufacture of hundreds of commercial products, and their industrial and consumer applications are expected to increase the chances of their exposure to the public [10, 11]. In particular, Cu-based NPs are widely used in a variety of established and emerging technologies, including catalysts, solar energy conversion, and antimicrobial agents, because of their distinct thermophysical properties and antimicrobial activities [16–20]. Despites widespread applications and the growing presence of Cu-containing nanoproducts, there is only limited information on the potential risks of exposure to Cu-based NPs compared to other NPs [17, 21]. To date, many reports regarding the in vitro toxicity of Cu-based NPs are available. Cu-based NPs induce cytotoxic effects associated with increase of reactive oxygen species in various cell lines, including human laryngeal epithelial cells and human alveolar type-I epithelial cells [22–25]. Cu NPs showed a higher toxicity than their oxide nanoparticles (CuO NPs) in HL60 cells [26]. CuO NPs are highly toxic compared to carbon nanotubes and other metal oxide NPs [27]. However, only a few studies have explored in vivo toxicity; these studies revealed biochemical and histological alterations related to liver, kidney, and spleen after a single or short-term exposure of Cu NPs [7, 15, 28, 29]. To our knowledge, there has been no report regarding the potential effects and biodistribution of Cu NPs following long-term exposure. Therefore, for further practical applications, it is necessary to evaluate the in vivo toxicity of Cu NPs and their biodistribution after subchronic exposure for the purpose of risk assessment.

Herein, we investigated the in vivo toxicity of Cu NPs and Cu microparticles (Cu MPs) following 28-day repeated oral dose in rats by evaluating biochemical, hematological, and histopathological parameters. The oral route was used because gastrointestinal exposure to nanomaterials has the potential for wide public exposure to higher doses and more frequent ingestion [30, 31]. Further, we investigated absorption, tissue distribution, and excretion to elucidate the main accumulation sites and elimination routes. In this work, we report for the first time, to the best of our knowledge, the in vivo toxicity and biodistribution of Cu NPs by conducting a repeated dose toxicity study.

## Results and discussion

### Physiochemical characterization of Cu NPs and Cu MPs

**Fig. 1:**
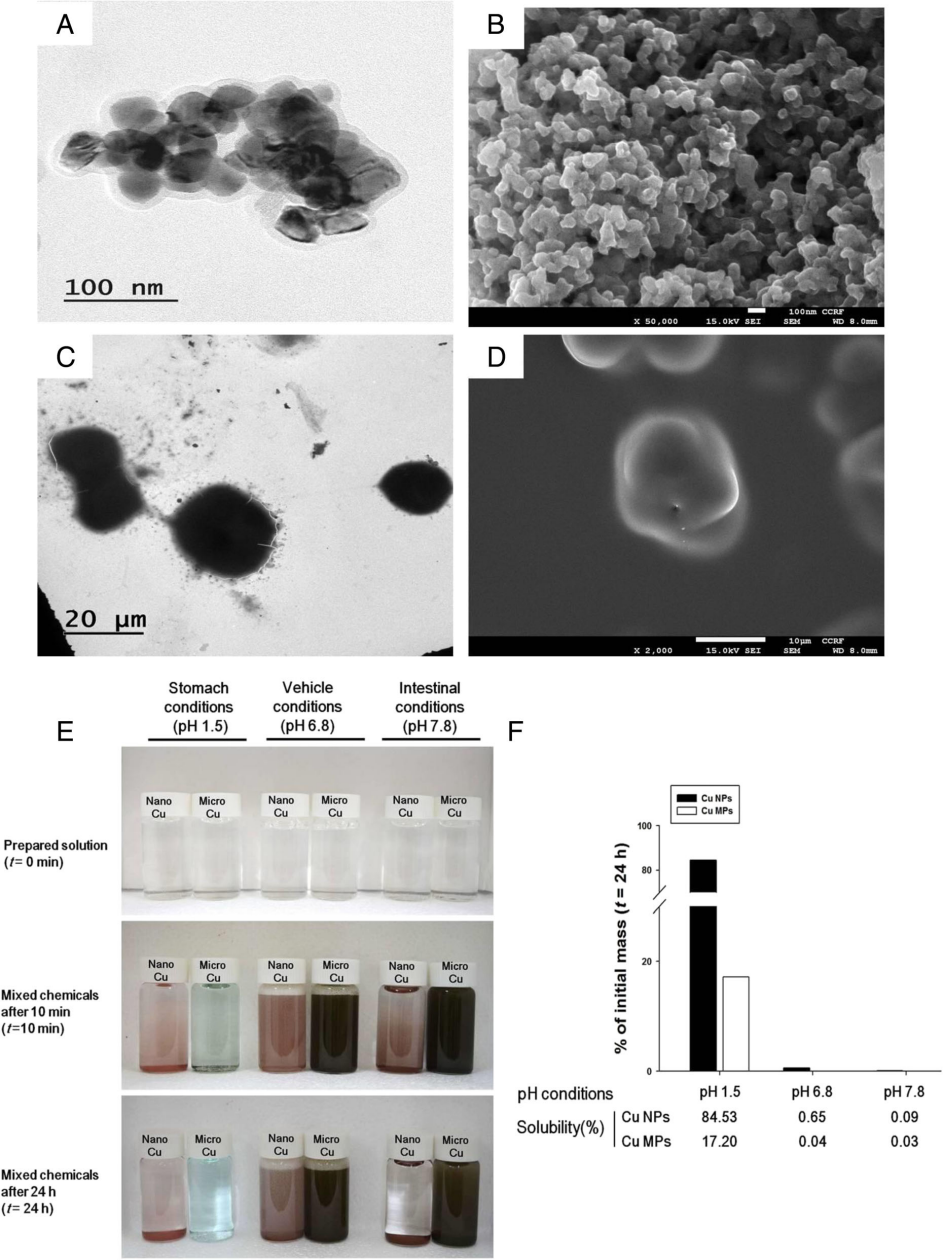
Morphology and dissolution of copper nanoparticles (Cu NPs) and copper microparticles (Cu MPs). Transmission electron microscopy (TEM) and scanning electron microscopy (SEM) images of (**a**, **b**) Cu NPs and (**c**, **d**) Cu MPs show generally spherical shape. **e** The color changes of artificial gastric fluid (AGF; pH 1.5), vehicle (1 % hydroxypropyl methylcellulose, HPMC; pH 6.5), and deionized water (pH 7.8) solution before (top, *t* = 0) or after mixing with the Cu NPs and Cu MPs for 10 min (middle, *t* = 10 min) and 24 h (bottom, *t* = 24 h). **f** Dissolution ratios of Cu NPs and Cu MPs in AGF, vehicle (pH 6.5), and deionized water (pH 7.8) milieus, which simulated in vivo physiological conditions. Cu NPs and Cu MPs (5 mg/mL) were suspended in above solutions and percentage of dissolution was measured using inductively coupled plasma mass spectrometry after 24 h of incubation (t = 24 h)

The physiochemical characteristics of Cu NPs and Cu MPs are summarized in Table 1. The morphology and actual size of Cu NPs and Cu MPs were characterized by transmission electron microscopy (TEM) and scanning electron microscopy (SEM). The morphology of NPs or MPs was spherical, and the actual size of individual particles was found to be 32.7 ± 10.45 nm (300 counts) and 25.3 ± 6.64 μm (100 counts), respectively (Fig. 1a–d). The purities of Cu NPs and Cu MPs were determined as 98.15 and 99.06 %, respectively, using energy dispersive X-ray spectroscopy (EDX) analysis on the same images (data not shown). The specific surface area of Cu NPs and Cu MPs was measured as 14.7 and 0.16 m^2^/g, respectively using the Brunauer-Emmett-Teller (BET) method. Suspension stability and surface charge reflect the interaction of NPs with physiological milieus because of their large surface area/volume ratio [32, 33]. The zeta potential of the Cu NPs was 25.5 ± 0.8 mV for pH 1.5, 1.32 ± 1.2 mV for pH 6.8, and -6.2 ± 0.2 mV for pH 7.8. Dynamic light scattering (DLS) measurements revealed that Cu NPs agglomerate in intestinal (pH 7.8) and vehicle (pH 6.8) milieus with the hydrodynamic diameters of 334.8 ± 128.26 and 516.4 ± 116.9 nm, respectively. However, in an acidic milieu (pH 1.5), the hydrodynamic diameter of Cu NPs was not determined because Cu NPs rapidly dissolved in acidic conditions and particle numbers were not enough to determine hydrodynamic diameter. These findings suggest the tendency of Cu NPs to aggregate/agglomerate in suspension or the intestinal milieu. However, the NP form presents a greater specific surface area compared with the same compositional particles of micro size scale that may cause different biological responses and modulate their fate in biological systems.

**Table 1:** Physiochemical characterization

Items		Cu NPs	Cu MPs
Shape		Spherical	Spherical
Primary size^a^		32.7 ± 10.5 nm	25.3 ± 6.6 μm
Hydrodynamic size (nm)^b^	pH 1.5	n.a	n.a
	pH 6.5	516 ± 116.9	n.a
	pH 7.8	334 ± 128.3	n.a
Surface area (m^2^/g)^c^		14.7	0.16
Zeta potential (mV)^d^	pH 1.5	25.5 ± 0.8	n.a
	pH 6.5	1.32 ± 1.2	n.a
	pH 7.8	−6.2 ± 0.2	n.a

Note

^a^Primary size of Cu NPs and Cu MPs was measured by transmission electron microscopy (300 counts and 100 counts, respectively)

^b^Hydrodynamic size of Cu NPs was measured by a dynamic light scattering method

^c^Surface area was measured by the Brunauer-Emmett-Teller method (N_2_ gas)

^d^Zeta potential was measured using electrophoretic light scattering method under pH 1.5, pH 6.5 and pH 7.8 conditions

*n.a*. not available

### Dissolution of Cu NPs and Cu MPs in physiological conditions

The dissolution of NPs in gastrointestinal fluids may help predict uptake and blood concentrations. The release of toxic ions has the potential to influence the toxicity of NPs [34–36]. Most NPs are rarely soluble at normal physiological conditions; however, their dissolution can be accelerated in acidic conditions [37, 38]. We evaluated the solubility of Cu NPs and Cu MPs (5 mg/mL each) under simulated gastric pH (pH 1.5), vehicle pH (pH 6.8), and intestinal pH (pH 7.8) after a 24 h incubation. Cu NPs in an acidic milieu showed bluish color changes within 10 min (Fig. 1e). After monitoring for up to 24 h, Cu NPs showed 84.5 % solubility in an acidic milieu, whereas only minimal dissolution was observed in vehicle (0.65 %) and intestinal (0.09 %) conditions (Fig. 1f). In contrast, Cu MPs showed much lower solubility compared to Cu NPs, even in an acidic milieu (pH 1.5, 17.2; pH 6.8, 0.04; and pH 7.8, 0.03 %). It has been reported that Cu NPs have high solubility in an acidic milieu with high positive charge at zeta potential analysis [15, 39]. Compared with Cu MPs, the large specific surface area of Cu NPs can lead to high reactivity and drastic interaction with hydrogen ions (H^+^) in a gastric milieu [15]. Because the pH of gastric fluid is between 1.5 and 2.0, Cu NPs may be dissolved in the stomach, and dissociated Cu ions can be absorbed into the systemic circulation when administered orally. The dissociation of Cu NPs after gastric emptying is prohibited in the basic milieu of the small intestine. Thus, the residence time in the stomach may influence the dissolution of Cu NPs. Cu NPs quickly dissolved in gastric milieus, and the undissolved NPs showed a delayed retention in the stomach after 24 h in mice exposed to Cu NPs, which may lead to durative interaction and persistent Cu ion generation in vivo [15].

### Absorption, distribution, and excretion of Cu

**Fig. 2:**
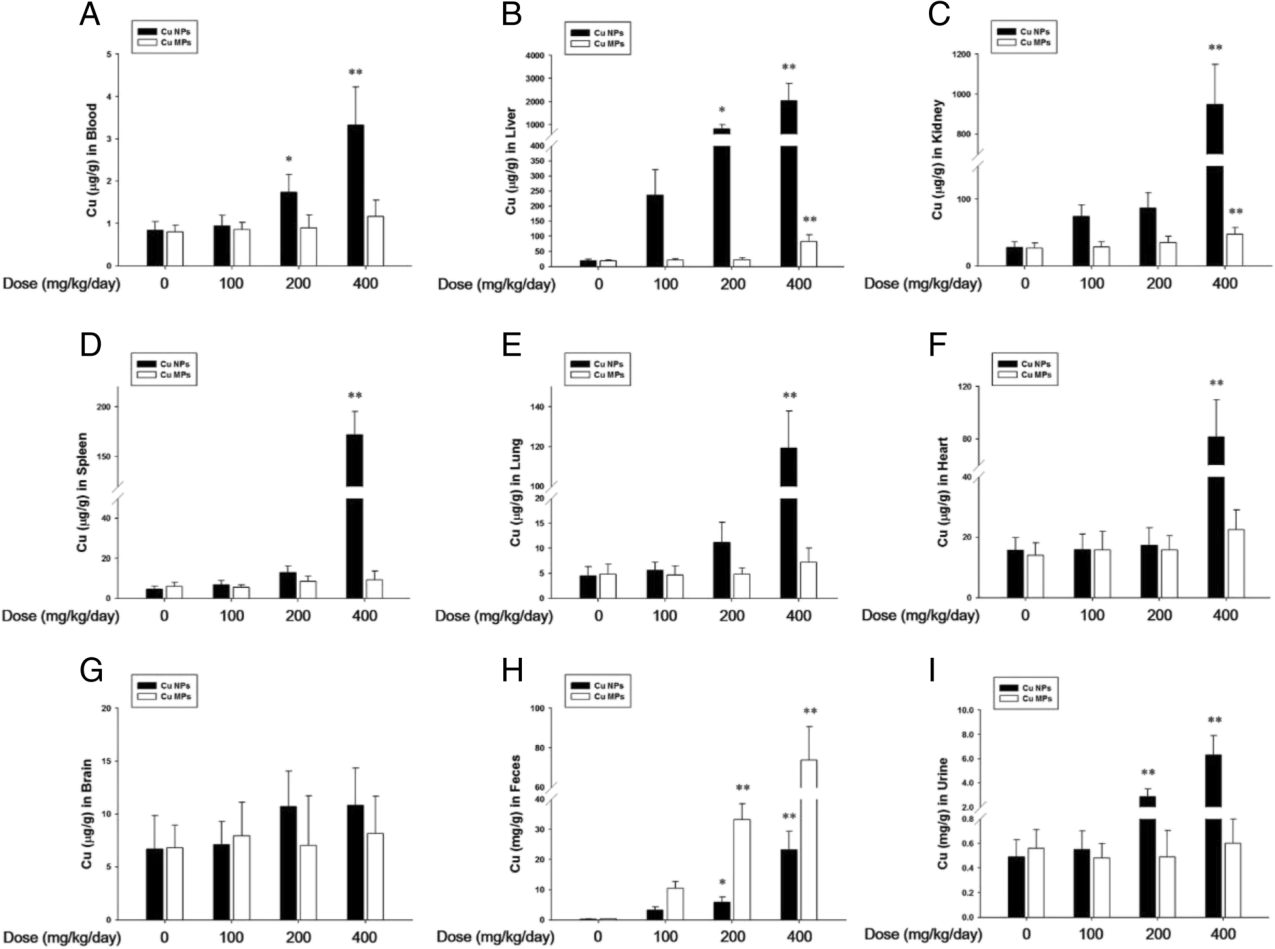
Absorption, biodistribution, and excretion of copper nanoparticles (Cu NPs) and copper microparticles (Cu MPs) following 4 weeks-repeated oral dose. **a** Systemic absorption of Cu was determined in blood Cu contents. **b**–**g** Biodistribution of Cu was determined in liver, kidney, spleen, lung, heart, and brain. The contents of Cu in (**h**) feces and (**i**) urine were used to determine excretion of Cu. The bar graphs present Cu contents of Cu NPs- and Cu MPs-treated rats at dose levels of 0, 100, 200, and 400 mg/kg/day, respectively (Cu NPs, black bars; Cu MPs, white bars). Values are presented as mean ± SD (*n* = 10). ^*, **^ *P* < 0.05, *P* < 0.01 versus vehicle control

The dissolution of NPs in physiological conditions and the physicochemical features of NPs are both likely to influence the absorption and biological response of NPs when administered orally [3, 35, 40]. The Cu levels in blood reflect the absorption of Cu following oral exposure of Cu NPs or Cu MPs. Cu levels in the blood of Cu NPs-treated rats showed a dose-dependent increase and were 4-fold higher than that of the vehicle control group (3.33 ± 0.89 μg/g vs. 0.83 ± 0.21 μg/g). In contrast, oral exposure to Cu MPs did not lead to an increase in blood Cu levels, which were not different from the vehicle control group (1.16 ± 0.30 μg/g vs. 0.83 ± 0.21 μg/g) (Fig. 2a). Exposure to nano-Cu (75 mg/kg) markedly elevates serum Cu level (3.5-fold higher) compared to a minimal increase in Cu from the same mass of micro-Cu exposed mice at 72 h after single oral dose [15]. Consistent with the results of a previous report, Cu NPs-treated rats showed 2.9-fold higher levels of Cu than that in rats exposed to a corresponding high dose of Cu MPs at 24 h after the last administration. With a delayed retention in the stomach, the higher levels of Cu in the blood of rats treated with Cu NPs compared to Cu MPs indicate that more Cu ions were dissociated from Cu NPs and absorbed into systemic circulation. Moreover, high and rapid dissolution of Cu NPs in the gastric milieu suggests that Cu NPs may be mainly absorbed as ionic forms rather than nanoparticulate states. Absorbed Cu ions could enter into systemic circulation and be distributed in various tissues. Generally, exposure of human body to Cu NPs can occur through different routes (e.g., inhalation, ingestion, injection or physical contact). Absorbed NPs may interact with biomolecules such as proteins, nucleic acids, lipids, and even biological metabolites [41]. Of particular importance is the absorption of proteins on the surface of NPs and form the NP-protein complexes, which referred to as the NP-protein corona. The protein corona alters the size and interfacial composition of NPs, giving it a new biological identity, which was determine the agglomeration, uptake, translocation, accumulation, as well as toxicological response [42]. Thus, further study to explanation for interaction between Cu NPs and proteins is needed.

In vivo biodistribution of Cu NPs can provide essential information regarding their accumulation sites. Cu levels in the tested organs of Cu NPs-exposed groups showed a dose-dependent increase when compared with that in the vehicle control group (Fig. 2b–g). The main organs with accumulation of Cu were liver (109-fold higher, 2038.1 ± 758.36 μg/g vs. 18.7 ± 5.79 μg/g), kidney (34-fold higher, 950.0 ± 197.58 μg/g vs. 27.7 ± 8.62 μg/g), and spleen (38-fold higher, 171.7 ± 23.64 μg/g vs. 4.5 ± 1.59 μg/g). Cu levels in nano-Cu treated mice showed a significant increase in the kidney, whereas micro-Cu did not elevate Cu levels in the kidney after a single oral dose (70 mg/kg) [15]. Feng et al. [43] reported that liver, spleen, lung, and kidney appear to be the major organs for accumulation of Cu sulfide nanoplates after intravenous injection. Thus, the biodistribution of Cu indicated that Cu from Cu NPs was mainly distributed in the liver, kidney, and spleen. Further, accumulated Cu in these organs can be a toxic reservoir based on their toxic potential. However, equivalent dose levels of Cu MPs are not only lower than those of Cu NPs, but also showed no dose-response increase in the tested organs, except in the liver (83.1 ± 20.94 μg/g) and kidney (46.7 ± 10.14 μg/g). This low distribution was due to the minimal absorption rate of Cu MPs.

The excretion of Cu was consistent with the absorption and distribution patterns of Cu NPs or Cu MPs. The levels of Cu in urine from the Cu NPs-treated group showed a significant increase with clear dose-response when compared to that in the vehicle control group (12.8-fold higher, 6.31 ± 1.59 μg/g vs. 0.49 ± 0.21 μg/g) (Fig. 2h). In contrast, only trace Cu levels were observed in the urine of the Cu MPs-treated groups (0.60 ± 0.19 μg/g vs. 0.49 ± 0.21 μg/g). Cu levels in the feces of Cu NPs treated rats showed clear dose-response when compared to that in the vehicle control group (115-fold higher, 26.2 ± 8.41 mg/g vs. 0.2 ± 0.09 mg/g). Cu MPs treated rats showed extremely high levels of Cu in the feces, which were 2.8-fold higher than that in Cu NPs treated-rats (73.7 ± 16.98 mg/g vs. 26.2 ± 8.41 mg/g) (Fig. 2i). Ingested Cu ions are mainly metabolized in the liver, and the major excretory route is via liver/bile [44]. The dissociation of Cu NPs after gastric emptying is prohibited in the basic milieu of the small intestine, and then unabsorbed NPs are excreted as feces [15]. Thus, extremely high levels of Cu in feces suggest that most of the absorbed Cu, dissociated from Cu NPs, or unabsorbed Cu NPs were predominantly excreted through feces; small amounts were excreted via the kidney/urine route. Most of the unabsorbed Cu MPs were also eliminated from gastro-intestinal tracts via the feces.

### Clinical signs, body weights, and food consumption

**Fig. 3:**
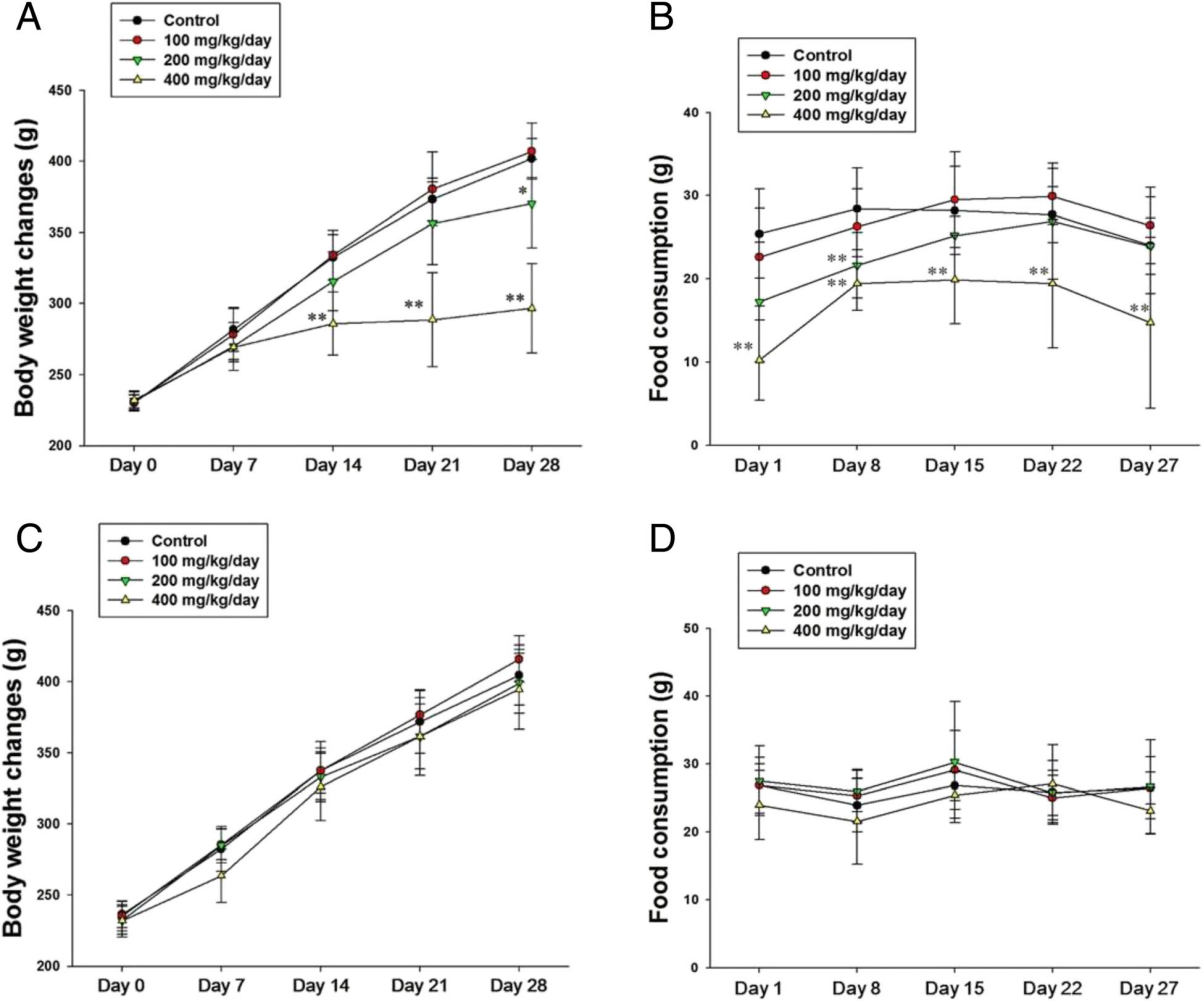
Body weight changes and food consumption of copper nanoparticles (Cu NPs)- and copper microparticles (Cu MPs)-treated rats following 4 weeks-repeated oral dose. Repeated oral dose toxicity of Cu NPs and Cu MPs was assessed by determining (**a**, **c**) Body weight changes and (**b**, **d**) food consumption of Cu NPs- and Cu MPs-treated rats. Values are presented as mean ± SD (*n* = 10). ^*, **^ *P* < 0.05, *P* < 0.01 versus vehicle control

Manifestations of toxicity, including anorexia, diarrhea, lethargy, and body weight loss, were observed in rats treated with Cu NPs [28]; these manifestations were similar to the effects of excessive Cu compound treatment [45–47]. In this study, treatment-related clinical signs observed in the high dose group of Cu NPs were consistent with toxic manifestations observed in a previous study. The body weight and the amount of food consumption in the high dose group of Cu NPs decreased significantly during the test period (Fig. 3a and b). In contrast, only test article-colored feces were observed in the high dose group of Cu MPs. The body weight and food consumption of the Cu MPs-treated groups showed no significant changes, even at the high dose, compared to that in the vehicle control group (Fig. 3c and d).

### Urinalysis, serum biochemistry, and hematology

**Fig. 4:**
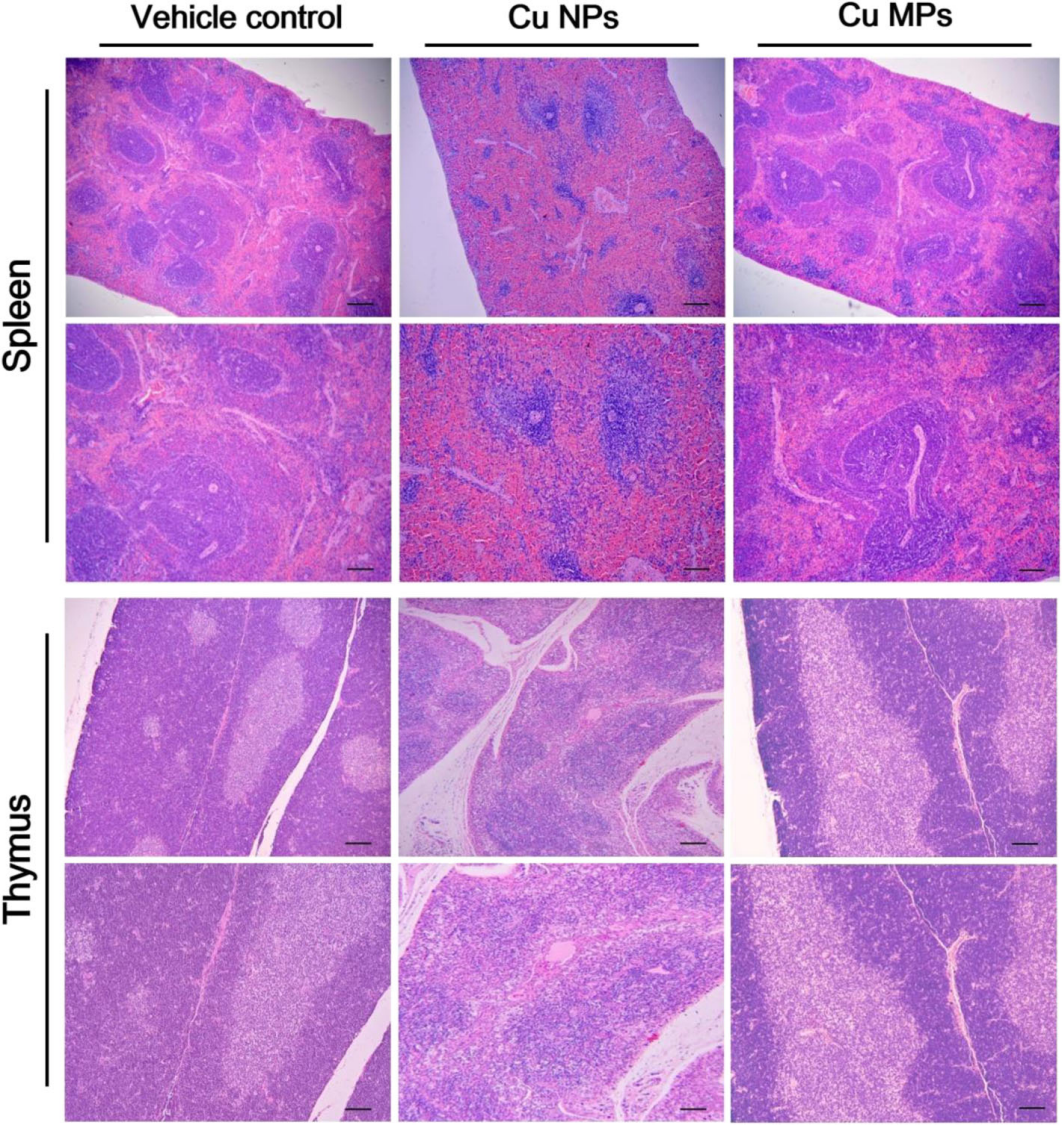
Histopathological changes in spleen and thymus of rats treated with copper nanoparticles (Cu NPs) and copper microparticles (Cu MPs) following 4 weeks-repeated oral dose. The rats treated with Cu NPs at 400 mg/kg/day showed moderate to severe degree of atrophic white pulp, decreased number of follicles and cellularity, and yellow pigmentation in spleen, disrupted demarcation of medulla/cortex, decreased cellularity of medulla/cortex, and cytoplasmic vacuolation in thymus. There were no changes in spleen and thymus from rats treated with Cu MPs. Hematoxylin and eosin stain

It has been reported that oral exposure to Cu NPs cause imbalance of acid and base by interacting with H^+^, resulting in metabolic alkalosis [15]. In a chronic metabolic alkalosis state, bicarbonate excretion ceased and led to a state of paradoxical aciduria [48]. The decreased urine pH observed in the high dose group of Cu NPs may be due to chronic metabolic alkalosis caused by sub-chronic exposure to Cu NPs (Additional file 1: Table S1). Other urinalysis parameters, including urine protein (PRO), occult blood (OB), leukocytes (LEU), specific gravity (SG), ketone body (KET), and nitrite (NIT), were increased significantly in the high dose group of Cu NPs. Hematological findings revealed that repeated exposure to Cu NPs resulted in red blood cell (RBC) destruction, which was characterized by a reduction of RBC, hemoglobin (HB), hematocrit (HCT), mean corpuscular volume (MCV), mean corpuscular hemoglobin (MCH), and mean corpuscular hemoglobin concentration (MCHC), as well as an increase in reticulocytes (RET) (Table 2). This interpretation was well supported by increased yellow pigmentation in the spleen. Chronic Cu intoxication causes hemolytic anemia with diverse hematological changes, including decreased RBC, HB, HCT, MCV, MCH and white blood cells (WBC) in rodents [46, 49, 50], which were consistent with the results of this study. In addition, the changes in the hematology indicate a microcytic anemia that generally observed with iron deficiency. Elevated levels of Cu levels have been shown to competitively inhibit iron absorption and utilization and to be correlated with diminution in serum iron levels [51, 52]. In the differential WBC count, a dose-dependent decrease in the percentage of lymphocytes (LYM) implied that Cu NPs might have adverse effects on the immune system, which was well correlated with the reduction of cellularity seen in the thymus and spleen (Fig. 4). The increased percentages of neutrophils (NEU) and monocytes (MON) were thought to be related to the inflammatory response of the affected organs and the decreased LYM percentage (Table 2). These results indicated that Cu NPs might affect red blood cells and immune organs (spleen and thymus). As reported previously, Cu NPs caused liver and kidney damages with biochemical alterations, including increased aspartate aminotransferase (AST), alanine aminotransferase (ALT), total bilirubin (TBIL), blood urea nitrogen (BUN), and creatinine (CRE) [28, 29]. With obvious changes in urinalysis parameters, Cu NPs treated rats showed a dose-related response in the increment of serum BUN, CRE, AST, ALT, TBIL, alkaline phosphatase (ALP), and lactate dehydrogenase (LDH), as well as the decrease of triglyceride (TG) and total protein (TP) with electrolytes disturbance (Table 3). These findings on the consequence of Cu NPs exposure proved that Cu NPs cause substantial damage to the liver and kidney. Collectively, Cu dissociated from Cu NPs mainly distributed into liver, kidney, and spleen, which caused obvious functional and structural damage. However, the parameters of urinalysis, serum biochemistry, and hematology were not affected by repeated exposure to Cu MPs (Additional file 1: Table S1, Tables 2 and 3).

**Table 2:** Hematological changes in male rats treated with Cu NPs and Cu MPs following 28 days-repeated oral dose

Items	Cu NPs (mg/kg/day)			
	0	100	200	400
No. of rats	10	10	10	10
RBC (10^12^/L)	7.02 ± 0.234^a^	6.69 ± 0.388	6.17 ± 0.346^**^	6.12 ± 0.612^**^
HB (g/dL)	16.78 ± 1.212	15.77 ± 1.668	12.70 ± 1.628^**^	11.80 ± 1.187^**^
HCT (%)	43.43 ± 3.435	42.50 ± 2.432	37.32 ± 3.179^**^	36.47 ± 5.747^**^
MCV (fl)	53.73 ± 4.388	50.09 ± 4.618	43.63 ± 9.582^**^	43.60 ± 4.486^**^
MCH (pg)	23.47 ± 1.598	21.31 ± 2.788	19.10 ± 3.306^**^	18.92 ± 2.099^**^
MCHC (g/dL)	34.68 ± 2.770	34.57 ± 3.317	33.05 ± 4.339	28.31 ± 2.198^**^
PLT (10^9^/L)	1106.2 ± 104.92	1212.7 ± 125.88	1258.8 ± 166.73^*^	1262.4 ± 121.65^*^
RET (%)	2.3 ± 0.23	2.4 ± 0.51	2.7 ± 0.48	6.1 ± 2.17^**^
WBC (10^9^/L)	11.2 ± 0.95	11.3 ± 2.13	8.5 ± 3.03^*^	8.3 ± 1.21^**^
NEU (%)	11.9 ± 0.95	11.2 ± 3.45	23.9 ± 5.41^**^	36.6 ± 8.15^**^
LYM (%)	82.3 ± 10.73	82.3 ± 12.79	71.7 ± 13.28	52.7 ± 20.17^**^
MON (%)	2.2 ± 0.39	2.2 ± 0.33	2.3 ± 0.51	3.0 ± 0.59^**^
EOS (%)	1.7 ± 0.39	1.7 ± 0.33	1.6 ± 0.53	2.0 ± 0.59
BAS (%)	0.7 ± 0.21	0.8 ± 0.18	0.7 ± 0.27	0.7 ± 0.19
LUC (%)	0.7 ± 0.13	0.7 ± 0.24	1.0 ± 0.19^*^	1.2 ± 0.25^**^
Items	Cu MPs (mg/kg/day)			
	0	100	200	400
No. of rats	10	10	10	10
RBC (10^12^/L)	7.04 ± 0.312	7.20 ± 0.286	7.00 ± 0.403	6.96 ± 0.388
HB (g/dL)	16.65 ± 1.326	16.72 ± 1.169	15.96 ± 1.563	16.12 ± 1.971
HCT (%)	43.79 ± 4.793	43.61 ± 4.201	40.16 ± 5.776	41.63 ± 3.304
MCV (fl)	53.81 ± 5.716	52.70 ± 6.621	52.63 ± 4.184	52.34 ± 7.206
MCH (pg)	23.61 ± 3.392	24.32 ± 2.562	24.52 ± 1.397	22.93 ± 4.257
MCHC (g/dL)	34.71 ± 3.392	29.67 ± 5.561	32.09 ± 3.397	32.25 ± 4.257
PLT (10^9^/L)	1179.7 ± 202.53	1047.4 ± 218.36	1259.8 ± 187.54	1236.5 ± 232.71
RET (%)	2.5 ± 0.58	2.6 ± 0.37	2.4 ± 0.39	2.7 ± 0.44
WBC (10^9^/L)	11.3 ± 1.49	11.9 ± 2.48	12.3 ± 3.49	11.56 ± 2.23
NEU (%)	12.2 ± 2.23	10.8 ± 3.91	13.2 ± 4.45	11.9 ± 3.71
LYM (%)	82.9 ± 13.2	85.0 ± 22.47	82.7 ± 16.66	83.5 ± 13.09
MON (%)	2.2 ± 0.48	2.3 ± 0.31	2.5 ± 0.43	2.3 ± 0.54
EOS (%)	1.6 ± 0.42	1.5 ± 0.24	1.6 ± 0.40	1.8 ± 0.48
BAS (%)	0.7 ± 0.23	0.7 ± 0.17	0.8 ± 0.13	0.7 ± 0.21
LUC (%)	0.7 ± 0.22	0.9 ± 0.29	0.9 ± 0.29	0.9 ± 0.23

Note. *RBC* red blood cell, *HB* hemoglobin, *HCT* hematocrit, *MCV* mean corpuscular volume, *MCH* mean corpuscular hemoglobin, *MCHC* mean corpuscular hemoglobin concentration, *PLT* platelet, *RET* reticulocytes, *NEU* neutrophils, *LYM* lymphocytes, *MON* monocytes, *EOS* eosinophils, *BAS* basophils, *LUC* large unstained cells

^a^Values are presented as mean ± SD

^*, **^ *P* < 0.05, *P* < 0.01 versus vehicle control group

**Table 3:** Serum biochemical changes in male rats treated with Cu NPs and Cu MPs following 28 days-repeated oral dose

Items	Cu NPs (mg/kg/day)			
	0	100	200	400
No. of rats	10	10	10	10
AST (IU/L)	115.6 ± 19.32^a^	125.2 ± 15.04	258.4 ± 21.52^**^	626.6 ± 60.04^**^
ALT (IU/L)	23.7 ± 2.80	33.3 ± 12.95	88.4 ± 20.65^**^	115.8 ± 19.12^**^
ALP (IU/L)	129.8 ± 12.45	132.0 ± 16.08	156.4 ± 22.06^*^	175.7 ± 38.23^**^
BUN (mg/dL)	19.4 ± 2.02	20.2 ± 3.24	22.4 ± 1.39	40.6 ± 6.72^**^
CRE (mg/dL)	0.23 ± 0.052	0.24 ± 0.041	0.26 ± 0.058	0.38 ± 0.068^**^
CPK (IU/L)	519.0 ± 124.43	504.2 ± 99.57	480.5 ± 94.72	335.2 ± 94.70^**^
TBIL (mg/dL)	0.25 ± 0.071	0.24 ± 0.055	0.34 ± 0.036	1.07 ± 0.318^**^
TCHO (mg/dL)	77.6 ± 8.82	79.7 ± 8.94	78.7 ± 2.52	68.6 ± 14.36
TG (mg/dL)	142.0 ± 25.52	133.4 ± 38.73	102.3 ± 29.10^*^	87.3 ± 28.43^**^
TP (g/dL)	6.2 ± 0.21	6.1 ± 0.31	5.4 ± 0.25^**^	5.4 ± 0.36^**^
ALB (g/dL)	4.2 ± 0.36	4.0 ± 0.12	4.0 ± 0.23	4.0 ± 0.17
LDH (IU/L)	227.7 ± 79.76	268.4 ± 55.99	321.1 ± 57.63^*^	462.3 ± 106.3^**^
Na (mEq/L)	141.8 ± 0.75	140.2 ± 1.35	140.3 ± 1.21	137.8 ± 2.95^**^
Cl (mEq/L)	97.6 ± 2.34	96.0 ± 1.17	95.8 ± 1.53^*^	93.4 ± 1.14^**^
K (mEq/L)	4.5 ± 0.49	4.8 ± 0.28	5.1 ± 0.26	6.0 ± 1.04^**^
Items	Cu MPs (mg/kg/day)			
	0	100	200	400
No. of rats	10	10	10	10
AST (IU/L)	113.7 ± 13.25	135.4 ± 29.86	138.6 ± 22.51	138.3 ± 21.89
ALT (IU/L)	24.9 ± 7.91	27.3 ± 10.83	24.7 ± 4.28	24.8 ± 5.69
ALP (IU/L)	124.7 ± 14.40	147.2 ± 20.99^*^	120.4 ± 18.95	122.8 ± 11.17
BUN (mg/dL)	17.3 ± 2.94	21.5 ± 4.85	21.5 ± 4.72	19.4 ± 3.67
CRE (mg/dL)	0.23 ± 0.045	0.26 ± 0.062	0.27 ± 0.058	0.24 ± 0.056
CPK (IU/L)	534.5 ± 106.82	522.7 ± 141.61	559.9 ± 80.12	516.9 ± 121.53
TBIL (mg/dL)	0.23 ± 0.045	0.24 ± 0.062	0.26 ± 0.058	0.25 ± 0.056
TCHO (mg/dL)	80.4 ± 6.41	77.7 ± 10.74	89.9 ± 15.03	77.7 ± 4.37
TG (mg/dL)	132.3 ± 20.45	139.9 ± 20.17	149.4 ± 24.16	137.6 ± 20.76
TP (g/dL)	6.4 ± 0.31	6.3 ± 0.47	6.1 ± 0.50	6.2 ± 0.16
ALB (g/dL)	4.2 ± 0.17	4.2 ± 0.27	4.3 ± 0.22	4.2 ± 0.22
LDH (IU/L)	256.8 ± 73.56	236.9 ± 61.90	298.9 ± 94.33	356.5 ± 98.59^*^
Na (mEq/L)	140.3 ± 1.16	141.1 ± 1.97	143.2 ± 1.14	141.6 ± 1.65
Cl (mEq/L)	97.3 ± 1.42	98.9 ± 1.52	97.6 ± 1.65	99.0 ± 1.67
K (mEq/L)	4.6 ± 0.32	5.1 ± 0.67	4.5 ± 0.36	4.6 ± 0.44

Note. *AST* aspartate aminotransferase, *ALT* alanine aminotransferase, *ALP* alkaline phosphatase, *BUN* blood urea nitrogen, *CRE* creatinine, *CPK* creatine phosphokinase, *TBIL* total bilirubin, *TCHO* total cholesterol, *TG* triglyceride, *TP* total protein, *ALB* albumin, *LDH* lactate dehydrogenase, *Na* sodium, *Cl*chloride, *K* potassium

^a^Values are presented as mean ± SD

^*, **^ *P* < 0.05, *P* < 0.01 versus vehicle control group

### Histopathology and organ weight changes

**Fig. 5:**
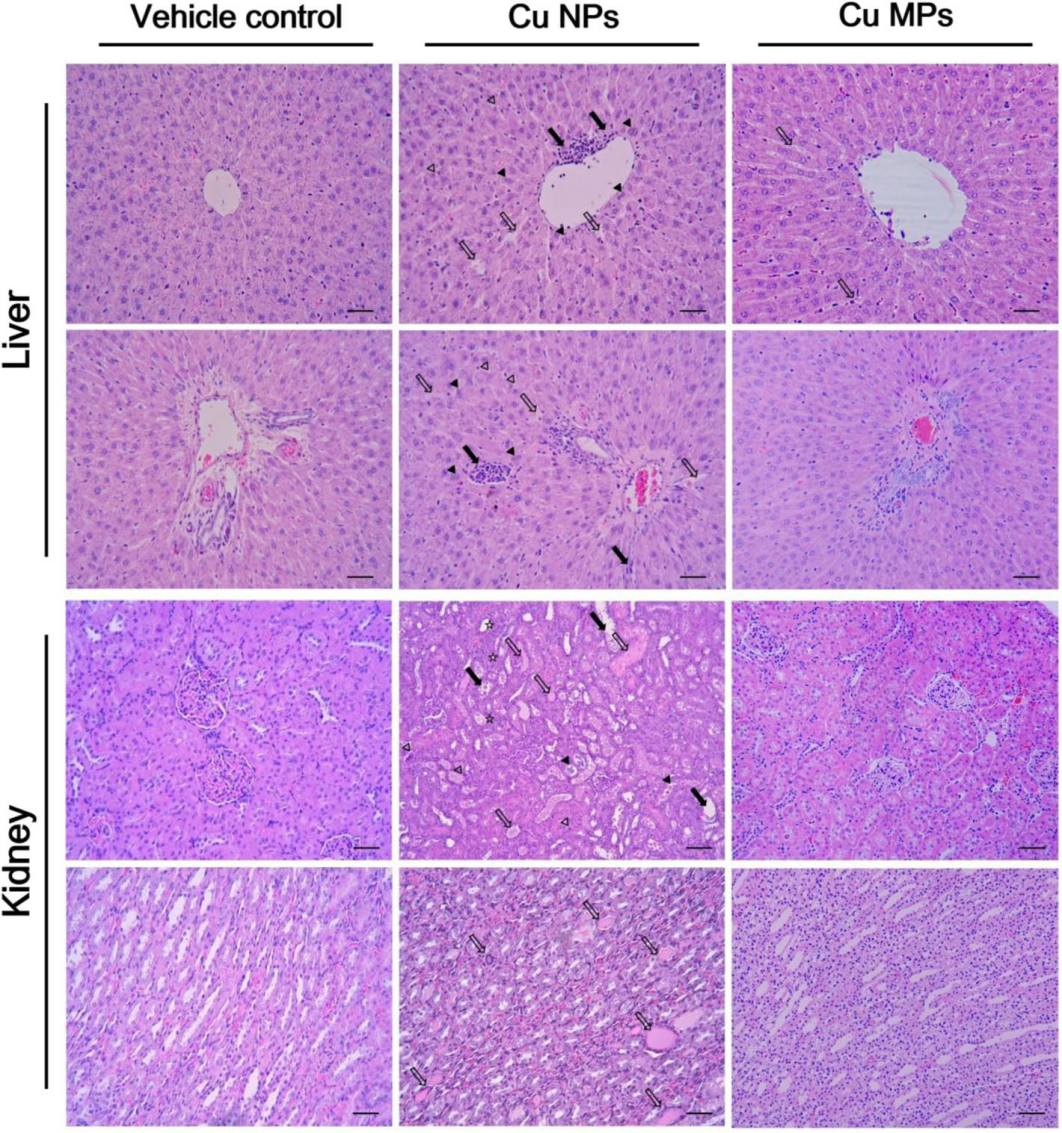
Histopathological changes in liver and kidney of rats treated with copper nanoparticles (Cu NPs) and copper microparticles (Cu MPs) following 4 weeks-repeated oral dose. Liver from rats treated with Cu NPs at 400 mg/kg/day showed mononuclear cell infiltration (*closed arrows*), dilated sinusoid (*open arrows*), degenerated hepatocytes (vacuolation; *open arrowheads*), and binucleated hepatocytes (*closed arrowheads*). The rats treated Cu MPs at 400 mg/kg/day showed only dilated sinusoid. Kidneys from rats treated with Cu NPs showed dilated tubules (*closed arrows*), cell debris in tubules (*closed arrowheads*), pink- or purple-colored cast in tubules (*open arrows*), degenerated tubular cells (*open arrowheads*), and inflammatory cell infiltration (*asterisks*). The rats treated with Cu MPs did not show changes in kidney structure. Hematoxylin and eosin stain

Our findings confirmed previous studies showing that a single or short-term oral exposure of Cu NPs induces severe damage to the kidney and liver [7, 15, 28, 29]. The major histopathological findings, including mononuclear cell infiltration, dilated sinusoid, degenerated or binucleated hepatocytes in the liver, dilated tubules, cell debris or pink or purple-colored casts in tubules, degenerated tubular cells, and inflammatory cell infiltration in the kidney, were observed in the rats treated with Cu NPs (Table 4 and Fig. 5). Excessive Cu intake results in impairment of both cellular and humoral immune responses [53]. Recently, Cu (II) chloride causes apoptosis of splenocytes and thymocytes, especially CD4^+^ T cell death [54, 55]. Exposure to nano-Cu caused dwindling of splenic units and reduction of lymphocytes [7]. In rats treated with the high dose of Cu NPs, the spleen exhibited atrophic white pulp, decreased number of follicles and cellularity, and yellow pigmentation, and the thymus displayed disrupted demarcation of medulla/cortex, decreased cellularity, and cytoplasmic vacuolation, which was consistent with the previous studies (Fig. 4). In particular, an apparent atrophic change of follicles (B cell area) and periarteriolar lymphoid sheath (T cell area) in the spleen and decreased cellularity in the cortex of the thymus were in agreement with the hematological findings of our study and the results of previous studies [7, 54, 55]. The changes in organ weight included increased kidney weight and decreased liver, spleen, and thymus weights in the high dose group of Cu NPs (Additional file 1: Table S2). These findings were of toxicological significance, because they were well supported by correlated biochemical, hematological, and histopathological changes. In contrast, Cu MPs-treated rats did not show obvious changes in histopathology and organ weights even at the high dose (Figs. 4, 5, and Additional file 1: Table S3). The remarkable reduction in prostate and seminal vesicle weights was observed at the high dose of Cu NPs. Chattopadhyay et al. [56] demonstrated that male rats treated with copper chloride at 2 mg/kg/day intraperitoneally for 26 days displayed adverse effects on testicular spermatogenesis and development of reproductive organs. Test substance-related stress in the toxicity study causes a decrease in the weights of reproductive organs, including epididymides, seminal vesicles, and prostates, but not in the testes [57]. Thus, further study is needed to determine the potential reproductive/developmental toxicity of Cu NPs because it is unclear whether decreased reproductive organ weights are related to the anti-androgenic effects of Cu dissociated from Cu NPs or to the stress-response phenomenon during the toxicity study.

**Table 4:** Histological changes in male rats treated with Cu NPs and MPs following 28 days-repeated oral dose

Items	Cu NPs (mg/kg/day)				Cu MPs (mg/kg/day)			
	0	100	200	400	0	100	200	400
No. of rats	10	10	10	10	10	10	10	10
Liver								
Mononuclear cell infiltration	-(0)^a^	-(0)	+(4)	++(8)	-(0)	-(0)	-(0)	-(0)
Dilated sinusoid	-(0)	+(2)	+(7)	+(9)	-(0)	+(2)	+(2)	+(4)
Degenerated hepatocytes	-(0)	-(0)	+(3)	+(9)	-(0)	+(1)	+(2)	+(1)
Binucleated hepatocytes	+(1)	+(1)	+(2)	+(7)	+(1)	+(2)	+(3)	+(1)
Kidney								
Dilated tubules	-(0)	+(2)	++(3)	++(8)	-(0)	+(1)	+(4)	+(3)
Cell debris in tubules	-(0)	-(0)	++(4)	++(8)	-(0)	-(0)	-(0)	-(0)
Purple-colored casts in tubules	-(0)	-(0)	+(5)	+++(7)	-(0)	-(0)	-(0)	-(0)
Degenerated tubular cells	-(0)	-(0)	+(2)	++(8)	-(0)	-(0)	-(0)	-(0)
Inflammatory cell infiltration	-(0)	-(0)	+(2)	+(7)	-(0)	-(0)	-(0)	-(0)
Spleen								
Atrophic white pulp	-(0)	-(0)	+(3)	++(7)	-(0)	-(0)	-(0)	-(0)
Decreased number of follicles	-(0)	-(0)	+(3)	+++(6)	-(0)	-(0)	-(0)	-(0)
Decreased cellularity	-(0)	-(0)	+(1)	++(7)	-(0)	-(0)	-(0)	-(0)
Yellow pigmentation	-(0)	-(0)	+(1)	+(5)	-(0)	-(0)	-(0)	-(0)
Thymus								
Decreased cellularity in medulla/cortex	-(0)	-(0)	+(2)	++(5)	-(0)	-(0)	-(0)	-(0)
Disrupted demarcation of medullar/cortex	-(0)	-(0)	+(2)	++(4)	-(0)	-(0)	-(0)	-(0)
Cytoplasmic vacuolation	-(0)	-(0)	-(0)	+(5)	-(0)	-(0)	-(0)	-(0)

^a^-, normal; +, mild; ++, moderate; +++, severe; (), number of case

Exposure to Cu NPs can occur through various routes (e.g., inhalation, ingestion, injection or physical contact). When administered orally, high solubility in the acidic milieu implies that Cu NPs can be dissociated into Cu ions in gastric pH conditions. Further, higher Cu levels in blood and tissues in rats treated with Cu NPs than Cu MPs indicate that absorbed Cu ions were distributed via circulation and accumulated in various tissues, which can be a toxic reservoir. Consistent with the above results, the toxicological study revealed that Cu NPs were more toxic than MPs of the same chemical composition at the same mass. The dissolution of Cu NPs may have an important role in their toxicity [7, 15]. Cu ion overload caused by excessive Cu NPs administration can cause damage to their accumulation sites, especially liver, kidney, and spleen [15, 43]. Additionally, the toxicity of CuO NPs was largely explained by soluble Cu ions [58]. Thus, the differences in dissolution play a crucial role in the gap of toxicological responses between Cu NPs and Cu MPs. In addition, the biopersistence of NPs influences long-term toxicity and is considered to be an important parameter needed for the risk assessment of NPs [36]. Therefore, further studies will be necessary to investigate whether the toxic responses of Cu NPs observed in this study are transient or persistent responses.

## Methods

### Test chemicals and preparation of test chemicals

Cu NPs (CAS No. 7440-50-8; 99.8 % purity) and Cu MPs (99 % purity) were purchased from SkySpring Nanomaterials (Houston, TX, USA) and Sigma-Aldrich (St. Louis, MO, USA), respectively. The information of particle size (measured by TEM) of Cu NPs and Cu MPs was 25 and 14–25 μm, respectively. Hydroxypropylmethylcellulose (HPMC, suspending vehicle) was purchased from Sigma-Aldrich. All other chemicals were of the highest grade commercially available. Test chemicals were dispersed into 1 % HPMC solution (w/v) with Milli-Q water. Particle suspensions were made fresh every day and prepared by ultrasonic dispersion (VCX130, Vibra Cell Sonics & Materials, Newtown, CT, USA) on ice for 20 min (130 W, 20 kHz, pulse 59/1) in agreement with recommendations by Taurozzi et al. [59].

### Physicochemical characterization and solubility of Cu NPs and Cu MPs

The primary size and morphology were measured by TEM (JEM-2100 F, JEOL, Tokyo, Japan) operating at 150 kV and SEM (Zeiss EVO-MA10, Carl Zeiss SMT, Cambridge, UK) operating at 15 kV. The purity of NPs and MPs was determined by EDX analysis on the same images from TEM (JEM-2100 F TEM equipped with X-Max^N^ 150 mm^2^ silicon drift detector; Oxford Instruments, UK). The samples for TEM were deposited on carbon-coated nickel grids and were air-dried overnight before analysis. The average size was obtained by measuring at least 100 particles using an image analyzer program (JEOL). The samples for SEM were dispersed on double-sided adhesive carbon tape onto an aluminum SEM stub, and then dusted to release loose particles. The specific surface area of NP and MP powder was measured by the nitrogen (N_2_) absorption based on the multipoint BET method using an ASAP2020 (Micromeritics, Norcross, GA, USA). In the solubility study, Cu NPs and Cu MPs were incubated under three physiological conditions: acidic conditions using artificial gastric fluid (AGF, pH 1.5), vehicle (1 % HPMC, pH 6.5), and basic conditions using deionized water (pH 7.8) for 24 h. AGF was prepared according to the previously described method [60]. In brief, 1.0 g NaCl (Affymetrix, Santa Clara, CA, USA) and 1.6 g pepsin (Sigma-Aldrich) were dissolved in 500 mL of DW and the pH of AGF was adjusted to 1.5 using 2 N HCl (Sigma-Aldrich). Deionized water (pH 7.8) was used to simulate the basic condition. Cu NPs and Cu MPs (5 mg/mL) were incubated in the above solutions for 24 h. NP- or MP-free supernatants were collected by three rounds of centrifugation at 150,000 × *g* for 30 min [37]. The samples weighing about 1 g were placed in 55 mL microwave digestion vessels and digested with 10 mL of concentrated nitric acid and 1 mL of 30 % H_2_O_2_ overnight. The samples were heated in a microwave digestion system (ETHOS One; Milestone, Sorisole, Italy). The microwave digestion system condition was 40 °C for 1 min, 100 °C for 20 min, and 170 °C for 2 h to remove the remaining nitric acid. Afterward, the samples were allowed to cool. After the samples were completely digested and colorless, the remaining solutions were diluted with 2 % nitric acid. The degree of ionization was evaluated by determining Cu^63^. Cu analysis of each sample was carried out using an ICP-MS method (NexION 300X, Perkin Elmer, Waltham, MA, USA). Cu standard solutions for ICP-MS calibration were prepared at concentrations of 5, 10, 50, and 100 ng/g. The fraction of solubilized Cu ions was calculated and expressed as a percentage by dividing the mass of Cu ions by the initial mass of Cu in Cu NPs or Cu MPs. The hydrodynamic diameter and zeta potential of NPs was measured by the DLS method using ELS-8000 (Otsuka Electronics, Tokyo, Japan) equipped with a 633 nm laser under above simulated physiological milieus.

### Animal handling and environmental conditions

Male Sprague–Dawley rats aged 7 weeks were obtained from a specific pathogen-free colony at Samtako Co. (Osan, Republic of Korea). The animals were acclimated for 1 week before starting the experiments. The body weight of the animals at the beginning of the study was (220 ± 19 g). Two rats per stainless wire mesh cage were housed in a room maintained at a temperature of 23 ± 3 °C and a relative humidity of 50 ± 10 % with artificial lighting from 08:00 to 20:00 and with 13 to 18 air changes per hour. Rats were provided tap water sterilized by ultraviolet irradiation and commercial rodent chow (Samyang Feed, Wonju, Korea) *ad libitum*. The Institutional Animal Care and Use Committee of Chonnam National University approved the protocols for the animal study (approval number: CNU IACUC-YB-2014-1), and the animals were cared for in accordance with the Guidelines for Animal Experiments of Chonnam National University.

### Experimental protocols and dose selection

The study was carried out in compliance with the Organization for Economic Cooperation and Development (OECD) test guideline TG407 for the testing of chemicals [61]. As males are more susceptible to the toxic effects of Cu NP than females [7, 15], we utilized male Sprague-Dawley rats for the in vivo toxicity study. A total of 80 healthy male rats were randomly assigned to eight experimental groups (*n* = 10). The test articles were administered by oral gavage to rats at dose levels of 100, 200, and 400 mg/kg/day, and two vehicle control groups were received 1 % HPMC alone. The experimental doses were selected based on the results of a preliminary dose-range finding study. Three groups of five male rats were exposed to Cu NPs via oral administration at doses of 50, 200, and 800 mg/kg/day for 2 weeks. At 800 mg/kg/day, the male rats displayed obvious general toxicity, such as suppressed body weight gain, decreased food intake, and various clinical signs, as well as death. At 200 mg/kg/day, Cu NPs produced a mild decrease in body weight gain and food intake. There were no treatment-related effects on clinical signs, body weights, or food intake at 50 mg/kg/day. On the basis of these results, 400 mg/kg/day was used as the high-dose, and the doses of 200 and 100 mg/kg/day were selected as mid- and low-doses, respectively, using a scaling factor of × 2. The dose levels of Cu MPs were also selected as 100, 200, and 400 mg/kg/day equivalent to the dose levels of Cu NPs for comparing the toxic effects and biodistribution. The administration volume (10 mL/kg body weight) of Cu NPs and Cu MPs was calculated based on the body weight of the individual animal measured each week. All animals were observed twice daily (before and after treatment) throughout the study period for any clinical signs of toxicity and mortality. The body weight of each rat and the level of food consumption were measured prior to the beginning of treatment and once a week during the experimental period. The amounts of food were calculated before they were supplied to the cages, and the remnants were measured the next day in order to calculate the difference, which was regarded as daily food consumption (g/rat/day). The weight gain was calculated by body weight on day 28 – body weight on day 0. The animals were sacrificed at 24 h (test day 28) after last administration of Cu NPs or Cu MPs.

### Urinalysis, hematology, and clinical chemistry

To collect urine and feces, six animals per groups were assigned to a metabolic cage for 6 h during the last week of the test period (test day 21). Urinalysis was carried out with fresh urine (2 mL per rats) within 1 h after collection to determine the urine levels of SG, pH, PRO, LEU, KET, OB, NIT, glucose, bilirubin, and urobilinogen by using the Multistix 10SG reagent strips and the Clinitek Status analyzer (Bayer Healthcare, Leverkusen, Germany). To collect blood samples, the animals underwent fasting overnight before scheduled necropsy (test day 28). During the scheduled necropsy, the blood samples (approximately 4 mL) were collected from the vena cava under carbon dioxide anesthesia. Approximately 1 mL of blood was collected in CBC bottles containing EDTA-2 K and analyzed within 1 h using an automatic hematology analyzer (Bayer ADVIA 120 Hematology Analyzer System, Leverkusen, Germany). Samples were analyzed for RBC (erythrocyte), HB, HCT, MCV, MCH, MCHC, platelets, WBC (leukocyte) count and the differential count of WBC. A portion of the blood (about 3 mL) was placed into tubes for serum separation and incubated at room temperature within 90 min. Serum samples were collected by centrifugation at 5000 × *g* for 10 min and evaluated with a blood chemistry autoanalyzer (Dri-chem 4000i, Fujifilm Co., Tokyo, Japan) for the following: AST, ALT, ALP, TP, BUN, CRE, TG, TBIL, glucose, albumin, total cholesterol, chloride, sodium, and potassium within 3 h after blood collection. After analysis of urine and blood, remaining urine and blood samples were stored immediately at −80 °C before Cu concentration analysis.

### Organ weights and histology

All organs were removed, weighed, and examined for macroscopically visible lesions. The weights of the following organs were measured: brain, thymus, heart, lung, liver, spleen, kidneys, adrenal glands, testes, seminal vesicles, prostates, and epididymides. The histopathological evaluation of organs and tissues was performed by fixing in a 10 % neutral-buffered formalin solution for 1 week. The tissues were stained with hematoxylin and eosin for microscopic examination. All observations were made manually in a blinded manner using a light microscope with × 5, ×10, ×20, and × 40 objective lenses and a × 100 oil immersion lens.

### *In vivo* absorption, distribution, and excretion of Cu from Cu NPs or Cu MPs

After The concentration of Cu in blood, tested organs (liver, kidney, spleen, heart, lung, and brain), urine and feces was determined by ICP-MS. Absorption of Cu in the Cu NP-treated rats was determined by using blood samples. To evaluate tissue distribution, tissue samples from the liver (left part of median lobe), spleen (left half), kidney (part of left kidney), lung (part of left lobe), heart (left half), and brain (left hemisphere) were obtained and weighed. Excretion of Cu was measured in urine and feces. The tissues, feces (about 0.3 g) or blood and urine (about 2 mL) samples were weighed and digested with concentrated nitric acid and 30 % H_2_O_2_ overnight. The samples were heated in a microwave digestion system (Milestone) at 170 °C to remove the remaining nitric acid until the samples were completely digested and till they became colorless. Finally, remaining solutions were diluted with 2 % nitric acid to a final acid concentration of 8–12 %. All samples were analyzed in duplicates for elemental Cu concentration (Cu^63^) using ICP-MS methods (Perkin Elmer, MA, USA).

### Statistical analysis

The numerical data were presented as means ± standard deviations (SD), and all statistical comparisons were analyzed by one-way analysis of variance (ANOVA) followed by Dunnett’s multiple comparison test. The urinalysis data were rank-transformed and analyzed by the non-parametric Kruskal–Wallis H-test. If a statistically significant difference was observed between groups, the Mann–Whitney *U*-test was used to identify the groups that were significantly different from the vehicle control group. A *P* value of < 0.05 was considered significant. Statistical analyses were performed using the GraphPad InStat v.3.0 (GraphPad Software, Inc., CA, USA).

## Conclusions

We described the in vivo toxicity and biodistribution of Cu NPs and Cu MPs following repeated oral exposure. The greater reactive surface area originating from its small size can lead to high reactivity, subsequently inducing rapid dissolution of Cu NPs in an acidic milieu. Thus, Cu NPs may be readily dissociated into their ionic forms in stomach compared with micro-size particles of the same composition. This is demonstrated by the Cu levels in blood and tested organs after Cu NP or Cu MP exposure. In vivo repeated dose toxicity study demonstrated that high surface area and high solubility could contribute to the toxicological responses of particles by causing Cu ion overload in their accumulation sites. Cu NPs affected RBC, liver, kidney, and immune organs (spleen and thymus), as well as male accessory reproductive organs at ≥ 200 mg/kg/day, whereas Cu MPs did not cause obvious changes at ≤ 400 mg/kg/day. Under these experimental conditions, the no-observed-adverse-effect level of Cu NPs and Cu MPs was considered to be 100 mg/kg/day and ≥400 mg/kg/day, respectively. In light of our findings, dissolution in physiological milieus influences absorption and biodistribution and acts as a determination factor in the toxic responses of particles in vivo when administered orally.

## Additional file

Additional file 1: Table S1. Urinalysis findings in male rats treated with Cu NPs or Cu MPs following 28 days-repeated oral dose. **Table S2.** Absolute and relative organ weights in male rats treated with Cu NPs following 28 days-repeated oral dose. **Table S3.** Absolute and relative organ weights in male rats treated with Cu MPs following 28 days-repeated oral dose. (DOCX 47 kb)

## Abbreviations

AGF: Artificial gastric fluid
ALP: Alkaline phosphatase
ALT: Alanine aminotransferase
AST: Aspartate aminotransferase
BET: Brunauer-Emmett-Teller
BUN: Blood urea nitrogen
CRE: Creatinine
Cu MPs: Copper microparticles
Cu NPs: Copper nanoparticles
CuO NPs: Copper oxide nanoparticles
DLS: dynamic light scattering
EDX: Energy dispersive X-ray spectroscopy
H^+^: Hydrogen ion
HB: Hemoglobin
HCO_3_^−^: Bicarbonate
HCT: Hematocrit
HPMC: Hydroxypropylmethylcellulose
ICP-MS: Inductively coupled plasma mass spectrometry
LDH: Lactate dehydrogenase
LEU: Leukocytes
MCH: Mean corpuscular hemoglobin
MCHC: Mean corpuscular hemoglobin concentration
MCV: Mean corpuscular volume
NIT: Nitrite
OB: Occult blood
PRO: Protein
RBC: Red blood cell
RET: Reticulocytes
SD: Standard deviation
SEM: Scanning electron microscopy
SG: Specific gravity
TBIL: Total bilirubin
TEM: Transmission electron microscopy
TG: Triglyceride
TP: Total protein
