# Supplementary material for: Comparative toxicity and biodistribution assessments in rats following subchronic oral exposure to copper nanoparticles and microparticles
Source: Part Fibre Toxicol. 2016 Oct 28;13:56. doi: 10.1186/s12989-016-0169-x (PMC5084351; doi:10.1186/s12989-016-0169-x)
Supplement: Additional file 1: Table S1. — Urinalysis findings in male rats treated with Cu NPs or Cu MPs following 28 days-repeated oral dose. Table S2. Absolute and relative organ weights in male rats treated with Cu NPs following 28 days-repeated oral dose. Table S3. Absolute and relative organ weights in male rats treated with Cu MPs following 28 days-repeated oral dose. (DOCX 47 kb) [file 12989_2016_169_MOESM1_ESM.docx]

**Table S1.** Urinalysis findings in male rats treated with Cu NPs or Cu MPs following 28 days-repeated oral dose

| Items | Grades | Scores | Cu NPs (mg/kg/day) | | | | Cu MPs (mg/kg/day) | | | |
| --- | --- | --- | --- | --- | --- | --- | --- | --- | --- | --- |
|  |  |  | 0 | 100 | 200 | 400 | 0 | 100 | 200 | 400 |
| No. of rats | |  | 6 | 6 | 6 | 6 | 6 | 6 | 6 | 6 |
| BIL | Negative | 0 | 6 | 6 | 5 | 5 | 6 | 5 | 6 | 5 |
|  | 1+ | 1 | 0 | 0 | 1 | 1 | 0 | 1 | 0 | 1 |
| KET | Negative | 0 | 5 | 5 | 2 | 1 | 5 | 5 | 6 | 4 |
|  | 1+ | 1 | 1 | 1 | 4 | 3 | 1 | 1 | 0 | 2 |
|  | 2+ | 2 | 0 | 0 | 0 | 2^*^ | 0 | 0 | 0 | 0 |
| SG | ≤1.015 | 0 | 0 | 0 | 0 | 0 | 0 | 0 | 0 | 0 |
|  | 1.020 | 1 | 0 | 0 | 0 | 0 | 1 | 0 | 0 | 0 |
|  | 1.025 | 2 | 5 | 4 | 4 | 0 | 4 | 4 | 5 | 4 |
|  | 1.030 | 3 | 1 | 2 | 2 | 1 | 1 | 1 | 1 | 2 |
|  | >1.030 | 4 | 0 | 0 | 0 | 5^**^ | 0 | 1 | 0 | 0 |
| pH | 6.0 | 0 | 0 | 0 | 0 | 0 | 0 | 0 | 0 | 0 |
|  | 6.5 | 1 | 0 | 0 | 0 | 2^**^ | 0 | 0 | 0 | 0 |
|  | 7.0 | 2 | 0 | 0 | 1 | 3 | 1 | 1 | 1 | 0 |
|  | 7.5 | 3 | 3 | 2 | 3 | 1 | 4 | 1 | 2 | 2 |
|  | 8.0 | 4 | 3 | 3 | 2 | 0 | 1 | 4 | 3 | 4 |
|  | 8.5 | 5 | 0 | 1 | 0 | 0 | 0 | 0 | 0 | 0 |
| PRO | Negative | 0 | 4 | 3 | 2 | 0 | 5 | 4 | 5 | 5 |
|  | 1+ | 1 | 2 | 3 | 4 | 1 | 1 | 1 | 1 | 1 |
|  | 2+ | 2 | 0 | 0 | 0 | 5^**^ | 0 | 1 | 0 | 0 |
|  | 3+ | 3 | 0 | 0 | 0 | 0 | 0 | 0 | 0 | 0 |
| OB | Negative | 0 | 6 | 4 | 4 | 0 | 6 | 5 | 6 | 5 |
|  | ± | 1 | 0 | 2 | 2 | 0 | 0 | 1 | 0 | 1 |
|  | 1+ | 2 | 0 | 0 | 0 | 2 | 0 | 0 | 0 | 0 |
|  | 2+ | 3 | 0 | 0 | 0 | 1 | 0 | 0 | 0 | 0 |
|  | 3+ | 4 | 0 | 0 | 0 | 3^**^ | 0 | 0 | 0 | 0 |
| LEU | Negative | 0 | 6 | 6 | 5 | 0 | 6 | 5 | 6 | 6 |
|  | ± | 1 | 0 | 0 | 1 | 1 | 0 | 1 | 0 | 0 |
|  | 1+ | 2 | 0 | 0 | 0 | 2 | 0 | 0 | 0 | 0 |
|  | 2+ | 3 | 0 | 0 | 0 | 2 | 0 | 0 | 0 | 0 |
|  | 3+ | 4 | 0 | 0 | 0 | 1^**^ | 0 | 0 | 0 | 0 |
| NIT | Negative | 0 | 6 | 6 | 6 | 1 |  | 6 | 6 | 6 |
|  | Positive | 1 | 0 | 0 | 0 | 5^**^ |  | 0 | 0 | 0 |

Note. BIL, bilirubin; KET, ketone; SG, specific gravity; PRO, protein; OB, occult blood; LEU, leukocyte; and NIT, nitrite.

^*,**^ *P* < 0.05, *P* < 0.01 versus vehicle control group

**Table S2.** Absolute and relative organ weights in male rats treated with Cu NPs following 28 days-repeated oral dose

| Items | Cu NPs (mg/kg/day) | | | |
| --- | --- | --- | --- | --- |
|  | 0 | 100 | 200 | 400 |
| No. of rats | 10 | 10 | 10 | 10 |
| Body weight at term | 402.1±13.82^a^ | 407.1±19.75 | 370.2±31.28^*^ | 296.6±31.50^**^ |
| Brain (g) | 1.93±0.114 | 1.88±0.093 | 1.90±0.142 | 1.84±0.116 |
| per b.w. (%) | 0.49±0.029 | 0.48±0.048 | 0.50±0.054 | 0.61±0.060^**^ |
| Liver (g) | 15.74±2.198 | 15.46±1.323 | 13.96±1.596 | 10.60±2.718^*^ |
| per b.w. (%) | 3.92±0.251 | 3.79±0.196 | 3.77±0.203 | 3.57±0.341^*^ |
| Spleen (g) | 0.97±0.142 | 0.89±0.091 | 0.88±0.076 | 0.66±0.074^**^ |
| per b.w. (%) | 0.24±0.023 | 0.22±0.017 | 0.23±0.026 | 0.21±0.016^**^ |
| Heart (g) | 1.26±0.104 | 1.24±0.048 | 1.25±0.126 | 0.99±0.074^**^ |
| per b.w. (%) | 0.32±0.029 | 0.31±0.013 | 0.33±0.021 | 0.33±0.029 |
| Thymus (g) | 0.56±0.142 | 0.56±0.161 | 0.52±0.128 | 0.30±0.147^**^ |
| per b.w. (%) | 0.14±0.014 | 0.14±0.020 | 0.14±0.018 | 0.10±0.021^**^ |
| Lung (g) | 1.56±0.213 | 1.49±0.214 | 1.48±0.378 | 1.44±0.323 |
| per b.w. (%) | 0.39±0.033 | 0.37±0.033 | 0.40±0.038 | 0.48±0.076^**^ |
| Kidneys (g) | 2.56±0.223 | 2.63±0.210 | 2.67±0.149 | 2.77±0.156^*^ |
| per b.w. (%) | 0.64±0.046 | 0.65±0.059 | 0.72±0.044^*^ | 0.94±0.086^**^ |
| Adrenal glands | 0.052±0.0078 | 0.052±0.0073 | 0.053±0.0028 | 0.054±0.0063 |
| per b.w. (%) | 0.013±0.0036 | 0.013±0.0059 | 0.014±0.0034 | 0.018±0.0044^*^ |
| Testes | 3.63±0.163 | 3.58±0.234 | 3.53±0.103 | 3.47±0.157 |
| per b.w. (%) | 0.90±0.084 | 0.88±0.102 | 0.95±0.095 | 1.17±0.168^**^ |
| Prostates (g) | 0.75±0.173 | 0.74±0.115 | 0.64±0.139 | 0.26±0.256^**^ |
| per b.w. (%) | 0.18±0.042 | 0.18±0.030 | 0.17±0.035 | 0.09±0.056^**^ |
| Seminal vesicles (g) | 1.73±0.226 | 1.58±0.340 | 1.46±0.356 | 0.67±0.695^**^ |
| per b.w. (%) | 0.43±0.065 | 0.38±0.059 | 0.39±0.073 | 0.24±0.149^**^ |
| Epididymides | 0.99±0.148 | 0.94±0.125 | 0.94±0.139 | 0.85±0.146^*^ |
| per b.w. (%) | 0.25±0.043 | 0.24±0.034 | 0.25±0.052 | 0.28±0.039 |

^a^ Values are presented as mean±SD.

^*, **^ *P* < 0.05, *P* < 0.01 versus vehicle control group

**Table S3.** Absolute and relative organ weights in male rats treated with Cu MPs following 28 days-repeated oral dose

| Items | Cu MPs (mg/kg/day) | | | |
| --- | --- | --- | --- | --- |
|  | 0 | 100 | 200 | 400 |
| No. of rats | 10 | 10 | 10 | 10 |
| Body weight at term | 404.7±21.1^a^ | 406.1±16.2 | 399.0±21.1 | 404.8±17.7 |
| Brain | 1.97±0.131 | 2.05±0.095 | 1.99±0.105 | 2.00±0.131 |
| per b.w. (%) | 0.48±0.037 | 0.51±0.025 | 0.49±0.053 | 0.49±0.046 |
| Liver | 16.14±1.891 | 15.97±1.258 | 15.64±1.142 | 15.55±1.005 |
| per b.w. (%) | 3.98±0.326 | 3.93±0.289 | 3.91±0.296 | 3.84±0.167 |
| Spleen | 0.99±0.091 | 0.97±0.082 | 0.95±0.079 | 0.99±0.089 |
| per b.w. (%) | 0.25±0.032 | 0.24±0.023 | 0.24±0.026 | 0.25±0.030 |
| Heart | 1.31±0.124 | 1.29±0.062 | 1.25±0.153 | 1.33±0.111 |
| per b.w. (%) | 0.32±0.028 | 0.32±0.035 | 0.31±0.032 | 0.33±0.025 |
| Thymus | 0.57±0.142 | 0.56±0.064 | 0.59±0.100 | 0.58±0.053 |
| per b.w. (%) | 0.14±0.024 | 0.14±0.015 | 0.15±0.025 | 0.14±0.015 |
| Lung | 1.59±0.231 | 1.52±0.190 | 1.67±0.311 | 1.56±0.199 |
| per b.w. (%) | 0.39±0.033 | 0.37±0.033 | 0.41±0.169 | 0.38±0.042 |
| Kidneys | 2.58±0.223 | 2.61±0.249 | 2.51±0.354 | 2.64±0.317 |
| per b.w. (%) | 0.63±0.064 | 0.64±0.061 | 0.63±0.083 | 0.65±0.042 |
| Adrenal glands | 0.054±0.0078 | 0.051±0.0178 | 0.051±0.0070 | 0.065±0.0115 |
| per b.w. (%) | 0.013±0.0036 | 0.013±0.0047 | 0.013±0.017 | 0.016±0.0031 |
| Testes | 3.58±0.263 | 3.49±0.230 | 3.57±0.213 | 3.64±0.359 |
| per b.w. (%) | 0.88±0.079 | 0.86±0.047 | 0.89±0.096 | 0.90±0.103 |
| Prostates | 0.73±0.173 | 0.76±0.113 | 0.66±0.065 | 0.74±0.100 |
| per b.w. (%) | 0.18±0.034 | 0.17±0.027 | 0.16±0.014 | 0.18±0.025 |
| Seminal vesicles | 1.63±0.226 | 1.68±0.314 | 1.50±0.396 | 1.48±0.262 |
| per b.w. (%) | 0.40±0.058 | 0.41±0.083 | 0.38±0.100 | 0.37±0.075 |
| Epididymides | 1.03±0.136 | 0.98±0.094 | 0.98±0.125 | 1.06±0.233 |
| per b.w. (%) | 0.25±0.036 | 0.24±0.027 | 0.25±0.036 | 0.26±0.048 |

^a^ Values are presented as mean±SD.
